# Amitraz Poisoning Treatment: Still Supportive?

**Published:** 2011

**Authors:** Nastaran Eizadi-Mood, Ali Mohammad Sabzghabaee, Farzad Gheshlaghi, Ahmad Yaraghi

**Affiliations:** a *Department of Poisoning Emergencies, Noor General Teaching Hospital, Isfahan University of Medical Sciences.*; b *Isfahan Clinical Toxicology Research Center, Isfashan University of Medical Sciences, Isfahan, Iran.*

**Keywords:** Amitraz, Bradycardia, Miosis, Central nervous system, Isfahan

## Abstract

Amitraz is a triazapentadiene, an α_2_ adrenergic agonist and a member of the amidine chemical family. A limited number of human intoxication cases have been published in the literature. Lack of a clear and specific protocol for the therapy of amitraz intoxication may make its successfully managed case reports useful and valuable for other clinical practitioners in poisoning departments.

The case is about a 22 years old female, single, university student, ingested a glass of amitraz poison (about 100 mL of a 20% solution) as a suicidal attempt on 11:30 am which was about 3.5 h before her hospital admission. She found nausea, vomiting, and dizziness. Immediately, her family took her to a clinic near their house. At that clinic (13:30 pm) she had miosis and they did gastric lavage , one adult dose of activated charcoal (50 g) and referred her to our Poisoning Emergency Department, where she was managed supportively and successfully.

Amitraz is a poisonous chemical which may cause central nervous system depression and also respiratory/cardiovascular symptoms as well. Several studies reported that using atropine for those amitraz poisoned patients with both miosis and bradycardia resolved the problem and recommend it as the first line of drug therapy when bradycardia occurs from vagal stimulation and atrioventricular block.

Management of amitraz poisoning is still considered to be supportive and symptomatic. Although the effects of activated charcoal and cathartics have not been studied, they may still be considered for treatment.

## Introduction

Amitraz is a triazapentadiene compound, a member of the amidine chemical family (1). It is a member of formammidine pesticides and is used worldwide (1). Amitraz is used as an insecticide/acaricide for controling the ectoparasites in animals (2). Commercial formulations of amitraz generally contain 12.5-20% of the drug in organic solvents, especially Xylene, which is also used as a solvent in paints, cleaners, and glues (3). 

Adverse reaction and side effects have been reported in animals exposed to the product, but only a limited number of human intoxication cases have been published in the literature. Amitraz is an α_2_ adrenergic agonist (1-3). It stimulates α_2 _adrenergic receptor sites in the central nervous system (CNS) and α_1_ adrenergic and α_2_ adrenergic receptor sites in the periphery (4). It also inhibits monoamine oxidase (MAO) enzyme activity and prostaglandin E_2_ synthesis (1). 

Amitraz poisoning may occur through the oral or dermal routes and potentially through inhaling (5). Poisoning is accompanied with numerous symptoms varying from central nervous system depression (drowsiness, coma, and convulsion), to miosis, or, rarely, mydriasis, respiratory depression, bradycardia, hypotension, hypertension, hypothermia or fever, hyperglycaemia, polyuria, vomiting, decreased gastrointestinal motility, and intestinal distension (1). 

## Experimental

A 22-year-old female, single, university student, ingested a glass of amitraz poison (20% solution) as a suicidal attempt on 11:30 am her first symptoms appear within an hour. At first, she had nausea, felt dizzy and vomiting followed afterward. Her family immediately took her to a clinic nearby. At that clinic they did gastric lavage and referred her to our Poisoning Emergency Department, in Noor Hospital, Isfahan, Iran. While being transferred to the hospital by emergency services, she lost her consciousness, had respiratory depression with shallow respiration, been intubated and received atropine because of bradycardia. On arrival to our center, her symptoms were coma with no response to painful stimuli, midriasis with negative light reflex. The vital signs were: (Blood pressure: 140/90), (Pulse rate: 84), (respiratory rate: 12), and (Temperature: 36.8°C). The analysis of blood gas was: (PaO_2_: 96.3), (O_2_ Saturation: 94), (pH: 7.15), (PCO_2_: 68.4) and (HCO_3_^-^: 23.5). Because of her shallow respiration and her ABG analysis, she required the mechanical ventilation support with SIMV mode. Furthermore, she received 50 g activated charcoal. Then the result of her ABG turned to: pH (7.44), PCO_2_ (34.2) and HCO_3_ (22.7). The other lab tests were:

BUN (13), Creatinine (0.9), Na^+^ (133; norm = 135-145), K^+^ (4.6), SGOT (61), SGPT (33), ALK. Phosphatase (182), Blood glucose (95 mg/dL; norm = 70-110 mg/dL), PT (13), PTT (40) and CBC (WBC: 9400, HCT: 40). Chest X-Ray was normal. We consulted with the neurologist, and everything seemed normal.

The following day at 5:00 am, her consciousness improved and she was extubated. At 8:00 am she gained her consciousness back and was able to answer to the questions. And finally, she completely recovered and discharged from the hospital in the afternoon.

## Results and Discussion

Amitraz is a pharmaceutical, veterinary, and an agricultural product which is used worldwide. It can cause poisoning in animals and humans when ingested, inhaled, or after skin exposure (1). 

Central nervous system depression was the predominant sign in our case, constant with the effect of amitraz on α_2_-adrenergic receptors. The observation of respiratory depression concomitant with central nervous system depression may suggest a direct inhibitory effect of the agent on the respiratory center (6). The sedative effects of α_2_-agonists are dose dependent (7). Presence of coma, absence of light reflex, and respiratory failure are probably due to the ingestion of a greater amount of amitraz, which supports its dose-dependent effects on body systems. Our patient got conscious after 20 h. The resolution time for CNS depression was reported to be 2-48 h in the previous reports (1-10). 

The α_1_– and α_2_-agonistic action of amitraz leads to bradycardia (1). In our case, bradycardia was also present, and miosis accompanied it. The co-existence of bradycardia and miosis and the respiratory depression may lead to confusion with some poisoning such as organophosphate or opioid poisoning; and both that are diagnosed should be excluded(11). Observation of miosis early in the intoxication followed by progression to mydriasis suggests that the presynaptic effect is dominant in the early phase and the postsynaptic effect in the late phase of the intoxication.

In our study atropine was used before reaching hospital to treat bradycardia. Using atropine for treatment of bradycardia is controversial (1, 5, 8, 12). However, most studies reported that using atropine for those with both miosis and bradycardia resolved the problem (8-10). Atropine is a first line therapy for the bradycardia that results from vagal stimulation and atrioventricular blocks, but not for those related to other mechanisms (13). The α_2_ adrenergic drugs can cause bradycardia by stimulating the dorsal motor nucleus of the vagal nerve (14). Hsu and colleagues claimed that atropine increased heart rate and prevented amitraz induced bradycardia in animals (15). We conclude that using atropine is effective when there is only bradycardia in amitraz poisoning. 

Although amitraz and its active metabolite inhibit insulin and stimulated glucagon secretion from the perfused rat pancreas in a concentration-dependent manner (16), we did not find hyperglycemia in our case.

Slight hyponatremia was observed in our case. The levels of BUN, creatinine, and the serum sodium and potassium usually do not change in amitraz poisoning (5). However kalyoncu and colleagues also reported hyponatraemia in their three cases (12). Rarely there is a minimal increase in the level of serum ALT and AST (5, 8). 

The analysis of blood gas showed respiratory alkalosis. The kalyoncu and colleagues reported respiratory alkalosis in two cases, respiratory acidosis in three cases, and metabolic acidosis in five cases (12). 

We did not observe any changes in her ECG. However in the study by Aydin and colleagues, non-specific ST changes were reported in the ECGs of seven children with no history of cardiac disease who recovered completely in 24 h (8). 

In conclusion, there is no specific antidote for amitraz poisoning and the management should be supportive and symptomatic. Although the effects of activated charcoal and cathartics have not been studied, they may still be considered for treatment (1). Particular attention must be given to monitoring and evaluating of the respiratory, cardiac, and central nervous systems. Since the sedative effects of α_2_ agonists are dose dependent, increased intake may lead to severe effects on the body systems causing coma and respiratory failure. The clinical presentations of our case were relatively severe and required intubation and mechanical ventilation. With the supportive management, the prognosis is good and the patients may be discharged healthy without any organ dysfunction. 

We believe that the action by producers, the regulatory authorities, and the national poisons control centers can minimize the amitraz poisoning. For example: the containers should be designed as childproof packages with striking and clear warning labels; the public education should be expanded on primary prevention of poisoning using media sources; and there should be new legislation for safety caps on poison containers (17).

## References

[1] Jorens PG, Zandijk E, Belmans L, Schepens PJ, Bossaert LL (1997). An unusual poisoning with the unusual pesticide amitraz. Hum. Exp. Toxicol.

[2] Queiroz-Neto A, Zamur G, Goncalves SC, Carregaro AB, Mataqueiro MI, Harkins JD (1998). Characterization of the antinociceptive and sedative effect of amitraz in horses. J. Vet. Pharmacol. Ther.

[3] Jones RD (1990). Xylene/amitraz: a pharmacologic review and profile. Vet. Hum. Toxicol.

[4] Cullen LK, Reynoldson JA (1990). Central and peripheral alpha-adrenoceptor actions of amitraz in the dog. J. Vet. Pharmacol. Ther.

[5] Aydin K, Kurtoglu S, Poyrazoglu MH, Uzum K, Ustunbas HB, Hallac IK (1997). Amitraz poisoning in children: clinical and laboratory findings of eight cases. Hum. Exp. Toxicol.

[6] Ulukaya S, Demirag K, Moral AR (2001). Acute amitraz intoxication in human. Intensive Care Med.

[7] Kambibayashi T, Atlee J (1999). Adreneroreceptor agonists. Complications in Anesthesia.

[8] Aydin K, Per H, Kurtoglu S, Poyrazoglu MH, Narin N, Aslan D (2002). Amitraz poisoning in children. Eur. J. Pediatr.

[9] Atabek ME, Aydin K, Erkul I (2002). Different clinical features of amitraz poisoning in children. Hum. Exp. Toxicol.

[10] Doganay Z, Aygun D, Altintop L, Guven H, Bildik F (2002). Basic toxicological approach has been effective in two poisoned patients with amitraz ingestion: case reports. Hum. Exp. Toxicol.

[11] Balali-Mood, M, K, Balali-Mood, Hosseini Shirazi (2006). Recent Advances In Treatment of Acute Organophosphorous Nerve Agents Poisoning. Iranian J. Pharm. Res.

[12] Kalyoncu M, Dilber E, Okten A (2002). Amitraz intoxication in children in the rural black Sea region: analysis of forty-three patients. Hum. Exp. Toxicol.

[13] Stancil, SA (2010). Case study: atropine and the bradycardia patient. Questioning the need for medical interventions is key to patient care. EMS Mag.

[14] Sanders KH, Jurna I (1985). Effects of urapidil, clonidine, prazosin and propranolol on autonomic nerve activity, blood pressure and heart rate in anaesthetized rats and cats. Eur. J. Pharmacol.

[15] Hsu WH, Lu ZX, Hembrough FB (1986). Effect of amitraz on heart rate and aortic blood pressure in conscious dogs: Influence of atropine, prazosin, tolazoline, and yohimbine. Toxicol. Appl. Pharmacol.

[16] Abu-Basha EA, Yibchok-Anun S, Hopper DL, Hsu WH (1999). Effects of the pesticide amitraz and its metabolite BTS 27271 on insulin and glucagon secretion from the perfused rat pancreas: involvement of [alpha] 2D-Adrenergic receptors. Metabolism.

[17] Ozanne-Smith J, Day L, Parsons B, Tibballs J, Dobbin M (2001). Childhood poisoning: access and prevention. J. Paediatr. Child Health.

